# Evaluation of motion symmetry in overweight cats with osteoarthritis in the stifle joint using a pressure sensitive mat technique

**DOI:** 10.1186/1751-0147-57-S1-P13

**Published:** 2015-09-25

**Authors:** Sarah Stadig, Anna Bergh

**Affiliations:** 1 Department of Clinical Sciences, Swedish University of Agricultural Sciences, Uppsala, Sweden; 2 Department of Anatomy, Physiology and Biochemistry, Swedish University of Agricultural Sciences, Uppsala, Sweden

## Introduction

An increasing number of cats are being recognized as overweight. Overweight is known to aggravate painful musculoskeletal conditions. Osteoarthritis (OA) is one of the most common musculoskeletal conditions that cause chronic pain in cats.

## Objectives

The objective was to assess the degree of motion symmetry in overweight cats with OA in the stifle joint using a pressure sensitive mat technique. The hypothesis was that cats with OA have a different motion symmetry index compared to healthy cats.

## Methods

Seven cats with OA in the stifle joint were included. The cats had signs of OA both on physical and radiographic examination. The cats were registered with a pressure mat (Walkway™ System High Resolution HRV4) and data analysis was performed on two valid trials. The results were compared to previous results from 24 healthy cats. The comparison was made using a two tailed t-test for unpaired data.

## Results

The cats with OA had a front/hind asymmetry of 1.42 compared to the healthy cats 1.26 (p=0.0012). The OA cats had an average bodyweight (BW) of 5.19 (standard deviation SD 0.51) and their body condition score (BCS) was 7.0/9. The healthy cats had an average BW of 4.71 (SD 1.45) and a BCS of 5.8/9.

## Discussion

This confirms the hypothesis that overweight cats with stifle joint OA have a different motion symmetry compared to healthy cats.

## Conclusion

Overweight cats with stifle joint OA put an increased amount of pressure on the front limbs compared to healthy cats.

